# A Facile Synthesis of New Monoazo Disperse Dyes Derived from 4-Hydroxyphenylazopyrazole-5-amines: Evaluation of Microwave Assisted Dyeing Behavior

**DOI:** 10.3390/molecules171213891

**Published:** 2012-11-23

**Authors:** Alya M. Al-Etaibi, Morsy A. El-Apasery, Maher R. Ibrahim, Nouria A. Al-Awadi

**Affiliations:** 1 Natural Science Department, College of Health Science, Public Authority for Applied Education and Training, P.O. Box 14281, Fayha 72853, Kuwait; 2 Chemistry Department, Faculty of Science, Kuwait University, P.O. Box 5969, Safat 13060, Kuwait; 3 Dyeing, Printing and Textile Auxiliaries Department, Textile Research Division, National Research Centre, Dokki, Giza 12622, Egypt

**Keywords:** aminopyrazoles, microwave irradiation, enaminone, disperse dyes, 2,4-pentanedione, enaminonitrile

## Abstract

A series of new monoazo disperse dyes containing pyrazolopyrimidine moieties was synthesized by coupling malononitrile or 3-aminocrotononitrile with 4-hydroxy- benzenediazonium chloride. Treatment of the resulting products with hydrazine hydrate yields the corresponding 4-arylazoaminopyrazoles, which then react with either 2,4-pentanedione and enaminonitriles or aryl-substituted enaminoketones to give the target pyrazolopyrimidine monoazo disperse dyes. Structural assignments of the dyes were made using both NMR spectroscopic and X-ray crystallographic methods. A high temperature dyeing method, by microwave irradiation, was employed with polyester fabrics. Most of the dyed fabrics tested displayed moderate light fastness and excellent washing and perspiration fastness levels.

## 1. Introduction

4-Arylazo-5-aminopyrazoles are readily obtainable, versatile compounds that have demonstrated antibiotic properties [1,2,3,4,5] and are used as dyes [6,7]. While a large number of arylazopyrazole dyes have been reported in the literature, very few condensed pyrazole derivatives carrying an arylazo function on the pyrazole ring have been reported [8,9,10,11,12,13]. The present study reports the synthesis of novel condensed 4-hydroxyphenylazopyrazolo[1,5-a]pyrimidine dyes, and their application as disperse dyes for polyester fabrics by a method using microwave irradiation as an energy source [14,15].

## 2. Results and Discussion

### 2.1. Synthesis

**Scheme 1:**
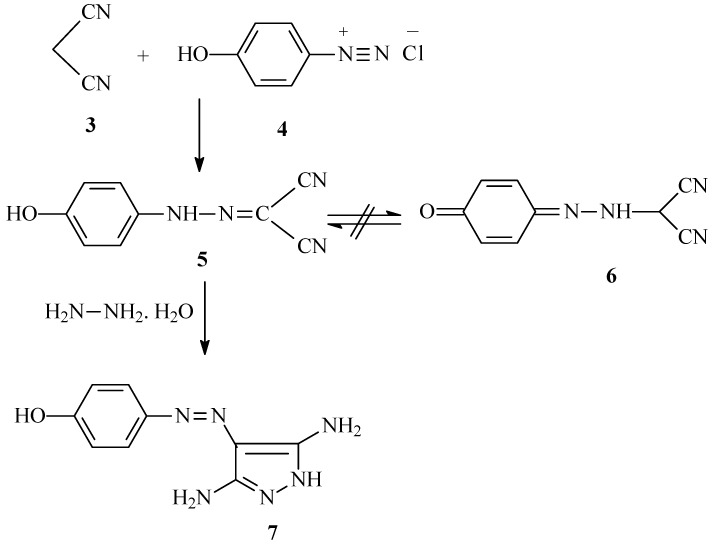
Synthesis of 4-(3,5-diamino-1*H*-pyrazol-4-ylazo)-phenol (**7**).

One of the sequences used for synthesis of the 4-hydroxyphenylazopyrazolo[1,5-a]pyrimidines is coupling of malononitrile (**3**) with *p*-hydroxybenzenediazonium chloride (**4**) to give arylhydrazone **5** (Scheme 1). ^13^C-NMR as well as NOE difference experiments show that this substance exists as phenol **5** rather than the cyclohexadienone tautomer **6**, as its ^13^C-NMR spectrum does not contain resonances for a sp^3^ hybridized carbon, NOE experiments show that irradiation of the OH proton signal at 9.63 ppm causes an enhancement of the aryl proton signal at 6.78 ppm and *vice versa*. Hydrazone **5** reacts smoothly with hydrazine hydrate to yield the diaminopyrazole **7**. It should be noted that although **7** was previously prepared using the same approach, evidence for its structural assignment was not provided in the earlier report [6].

**Scheme 2:**
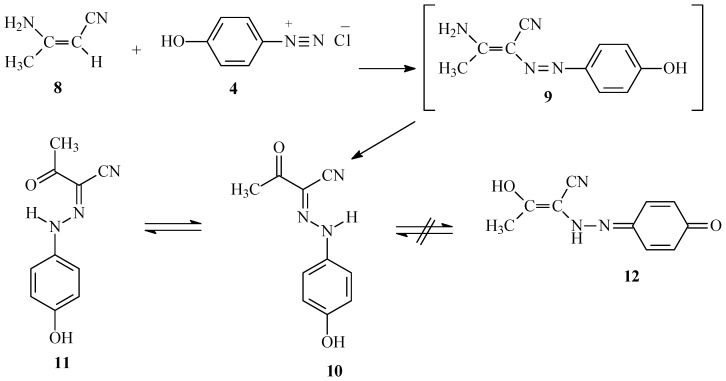
Synthesis of 2-[(4-hydroxyphenyl)-hydrazono]-3-oxo-butyronitrile (**10**).

Similarly, 3-aminocrotononitrile **8** undergoes coupling with diazonium salt **4** to yield the hydrazone **10** rather than the quinohydrazone **12**, *via* hydrolysis of the azo-intermediate **9** (Scheme 2). The possibility that **11 **exists in the *syn* form because of the hydrogen bonding stabilization is considered unlikely based upon previous studies that show stereoelectronic factors dominate in such systems [16].

NOE difference experiments were done to help establish the structure of **10**. The results indicate that irradiation of the NH signal at 12.1 ppm causes an enhancement of the intensities of the aryl proton resonances at 7.39 and 6.80 ppm. In addition, irradiating the OH signal at 9.58 ppm does not promote any enhancement of these signals.

**Scheme 3:**
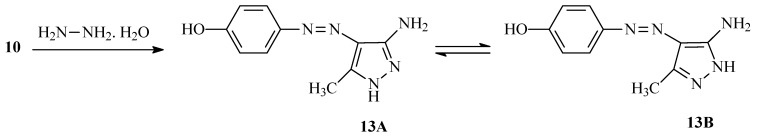
Synthesis of 4-(3-amino-5-methyl-1*H*-pyrazol-4-ylazo)-phenol (**13A**) and 4-(5-amino-3-methyl-1*H*-pyrazol-4-ylazo)-phenol (**13B**).

Reaction of hydrazone **10** with hydrazine hydrate (Scheme 3) generates the corresponding aminopyrazole that was found to exist as a 1:1 mixture of tautomers **13A** and **13B** according to a ^1^H-NMR experiment in DMSO-d*_6_* solution at room temperature. NOE difference experiments show that irradiation of the NH signal at 11.94 ppm corresponding to **13A** enhances the methyl proton signal at 2.36 ppm.

Pyrazoles **7** or **13** react with 2,4-pentanedione to yield 4-hydroxyphenylazopyrazolo[1,5-a]-pyrimidines **14a** and **14b**, both of which exist in their phenolic forms. This conclusion is also based on NOE difference experiments which demonstrate that irradiation of the OH signals at 9.78 and 9.96 ppm for **14a** and **14b** respectively, enhances the intensities of the respective *ortho* aryl proton signals 6.86 and 6.89 ppm.

**Scheme 4:**
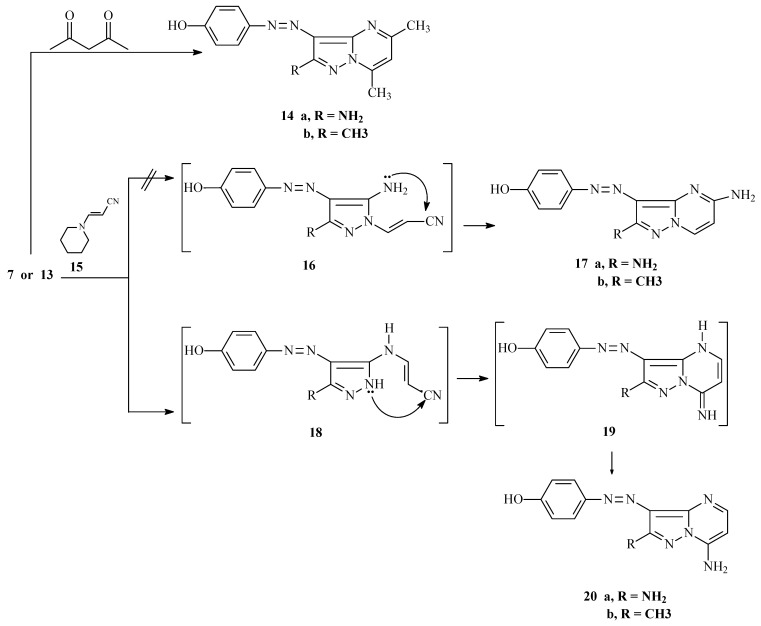
Synthesis of compounds **14a**,**b** and **20a**,**b**.

**Figure 1:**
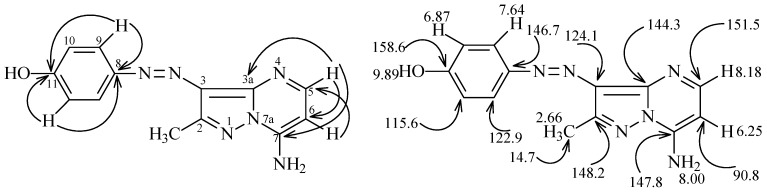
^1^H- and ^13^C-NMR resonance assignments of compound **20b**.

Similarly, **7** or **13** react with 3-piperidinylacrylonitrile **15** to produce the corresponding azopyrazolo[1,5-a]pyrimidines that might have either regioisomeric structure **17** or **20** (Scheme 4). The assignment of structures **20a **and **20b** was made by H-C correlations observed in HMBC 2-D experiments. The important HMBC correlations for **20b** (Figure 1) are: (**a**) H^5^ at 8.18 ppm with C^3a^, C^6^ and C^7^ at 144.3, 90.8 and 147.8 ppm, respectively; (**b**) H^6^ at 6.25 ppm with C^5^ at 151.5 ppm; (**c**) H^9^ at 7.63 ppm with C^8^ and C^11^ at 146.7 and 158.6 ppm, respectively; and (**d**) H^10^ at 7.87 ppm with C^8^, C^11^ at 146.7 and 158.6 ppm, respectively. Further information came from the results of ^1^H-^15^N HMBC experiments, which show that N^7a^ and N^4^ resonate at 205 and 235 ppm, respectively, this cross peak correlations exist for the shielded proton H^6^ at 6.25 ppm with N^7a^ at 205 ppm (*^3^J*) (H^6^, N^7a^), and that the deshielded proton H^5^ at 8.18 ppm with N^4^ at 235 ppm (*^2^J*) (H^5^, N^4^).

**Scheme 5:**
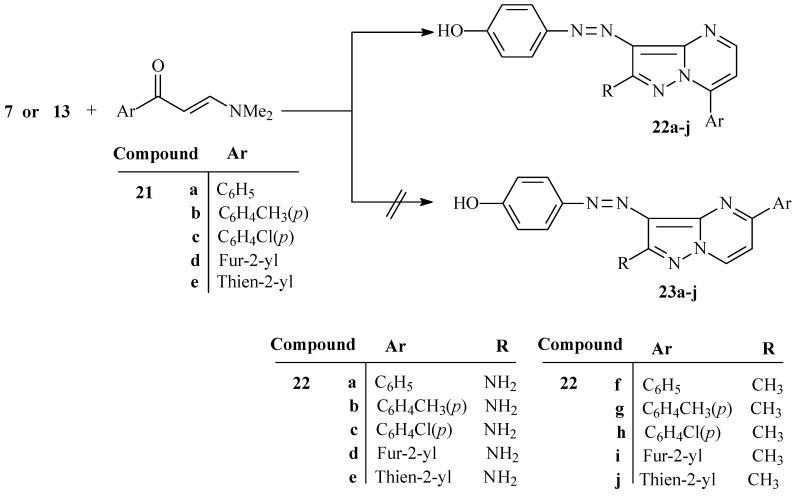
Synthesis of pyrazolo[1,5-a]pyrimidine disperse dyes **22a**–**j**.

Finally, the azopyrazoles **7** or **13** condense with enaminones **21a**–**e** to yield structures which might be formulated as either **22** or **23**. ^15^N-HMBC experiments for compound **22c** revealed that the chemical shifts for N^7a^ and N^4^ are 208.9 and 266.6 ppm, respectively, and that cross peak correlations exist for coupling of the shielded proton H^6^ at 7.24 ppm with N^7a^ at 208.9 ppm (*^3^J*) (H^6^, N^7a^) and N^4^ at 266.6 ppm (*^3^J*) (H^6^, N^4^), and for coupling of the deshielded proton H^5^ at 8.58 ppm with only N^4^ at 266.6 ppm (*^2^J*) (H^5^, N^4^) (Scheme 5). These observations demonstrate that the azopyrazolo[1,5-a]pyrimidines have general structures **22**.

**Figure 2:**
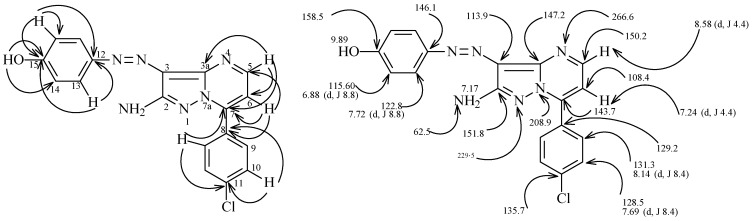
^1^H and ^13^C NMR resonance assignments of compound **22c**.

**Figure 3:**
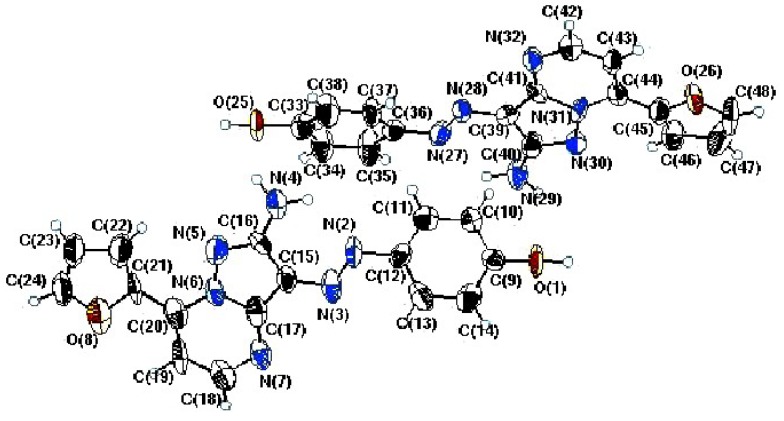
ORTEP plot of the x-ray crystallographic structure of **22i**.

To confirm this conclusion, 2D NMR experiments were performed for **22c**, giving the data displayed in Figure 2, and X-ray crystallographic analysis was done for **22i** (Figure 3) [17]. Selected bond distances, angles and structure refinement in the crystal structure are given in Table 1 and Table 2, the data clearly show that the presence of N=N arylazo moiety and that the 7a-nitrogen (N31 in Figure 3) has sp^2^ hybridized character.

In addition, the azo moiety has *E*-geometry, thus enabling hydrogen bonding interaction between a hydrogen of the amino group (N29) and azo nitrogen (N27). Finally, the entire molecule is nearly planar, indicating that all atoms comprising the basic structure are sp^2^ hybridized.

**Scheme 6:**
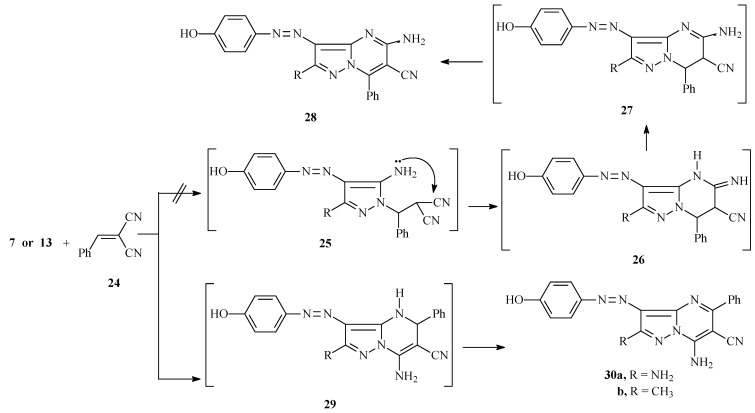
Synthesis of compounds **30a**,**b**.

It has been reported that reaction of the diaminopyrazole **7** with benzylidenemalononitrile **24** results in the formation of azopyrazolo[1,5-a]pyrimidines **28**, whose structural assignment was made based on the results of a previous investigation carried out by Elfahham *et al.* [18]. However, we observed that **7** reacts with **24** to yield the regioisomeric azopyrazolo[1,5-a]pyrimidines **30a**, whose structure was determined by using 2D NMR spectroscopic methods. Similarly the reaction of **24** with **13** afforded the corresponding azopyrazolo[1,5-a]pyrimidine **30b** (Scheme 6).

**Table 1:** Selected bond lengths and angles for **22i**.

Bond	Bond length (Å)	Bond	Bond angle (°)
N27—N28	1.286 (6)	N31—C44—C45	119.7 (6)
N31—N30	1.373 (6)	C36—N27—N28	114.3 (6)
N30—C40	1.345 (7)	N30—N31—C44	126.3 (6)
N32—C41	1.357 (6)	N31—C41—C39	103.7 (7)
N32—C42	1.324 (7)	C44—N31—C41	120.9 (6)
N27—C36	1.416 (7)	C44—N31—C41	120.9 (6)
N29—C40	1.345 (7)	C40—N29—H29A	119.9(5)
N31—C41	1.437 (7)	C40—N29—H29B	120.1(5)

**Table 2:** Crystal data and structure refinement for compound **22i**.

Chemical formula	C_16_H_12_N_6_O_2_	Z	4
**Formula weight**	320.312	**Temperature**	298 K
**Crystal System **	Triclinic	**Radiation type**	Mo *K*α
**Space group**	P-1	**Measured reflections**	5804
***a ***	8.4725 (4)Å	**Independent reflections**	6544
***b ***	8.5332 (5)Å	**Observed reflections**	1068
***c ***	22.111 (2)Å	**R_int_**	0.066
***α***	97.401 (3)°	**R(all) **	0.329
***β***	92.591 (3)°	**R(gt)**	0.054
***γ***	113.776 (7)°	**wR(ref)**	0.094
**V**	1442.4 (2)Å^3^	**wR(all)**	0.270
**λ**	0.71073	**Parameters**	433

**Figure 4:**
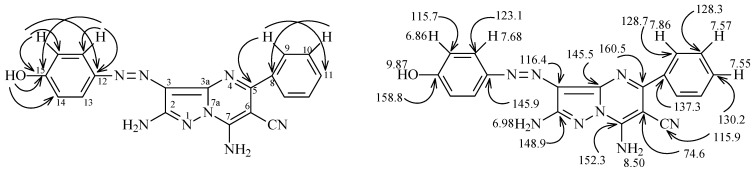
^1^H and ^13^C NMR signal assignments of **30a**.

The ^1^H- and ^13^C-NMR signal assignments and H-C correlations in the HMBC 2-D experiment of **30a** are displayed in Figure 4. Specific data from these measurements show that H^9^ at 9.87 ppm correlates with C^5^ and C^11^ at 160.4 and 130.2 ppm, respectively, H^10^ at 7.57 ppm correlates with C^8^ at 137.3 ppm, H^13^ at 7.68 ppm correlates with C^12^, C^13^ and C^15^ at 145.9, 123.0, 158.8 ppm, respectively, H^14^ at 6.86 ppm correlates with C^12^, C^14^ and C^15^ at 145.9, 115.8, 158.8 ppm, respectively, and OH at 9.87 ppm correlates with C^14^ and C^15^ at 115.8 and 158.8, respectively.

The regioisomerism assignment of **30a** was confirmed by comparison of the pyrimidine carbons of **30b** in the ^13^C-NMR which appear at almost the same positions at δ = 160.9, 74.3, 150.4 ppm respectively. The structure of **30b** was confirmed by ^15^N-HSQC and ^15^N-HMBC. Thus, ^15^N-HSQC shows the NH_2_ at δ = 87 ppm, and in ^15^N-HMBC the CH_3_ protons correlates with N^1^ at δ = 260 ppm and the NH_2_ protons correlates with N^7a^ at δ = 195 ppm (which is close to the N^7a^ chemical shift of **20b** and **22c**).

### 2.2. Dyeing and Fastness Properties

The 4-hydroxyphenylazopyrazoles **7**, **13**, and their pyrimidine derivatives **22a**–**d**, and **22f**–**h** were explored as dyes for polyester fabrics at 1%–6% shades using the high temperature dyeing method (HT) at 130 °C for 60 min with microwave heating as the energy source. The physical and analytical data for the dyed fabrics, given in Table 3 and Table 4, show that use of microwave irradiation leads to a large increase in dye uptake and dyeing rates along with enhancements in performances of dye leveling and color homogeneity as compared by conventional method.

**Table 3:** Color strengths of monoazo disperse dyes on polyester fabrics.

DyeNo	Molecular weight	Color shade on polyester	Color strength (K/S)% Dye o.m.f.					
			1	2	3	4	5	6
**7**	234	Yellowish brown	0.44	0.84	1.00	1.16	1.49	1.76
**22a**	330	Yellow	7.72	9.78	10.03	13.03	13.33	16.88
**22b**	344	Yellow	14.59	15.72	15.81	17.76	21.18	24.13
**22c**	364	Yellow	10.72	14.92	16.23	17.26	21.96	24.42
**22d**	320	Yellowish brown	15.44	21.08	23.02	25.30	26.25	28.07
**13**	217	Pale brown	2.18	4.35	4.88	6.43	12.86	13.10
**22f**	329	Yellowish orange	22.12	23.55	24.04	24.41	25.20	25.67
**22g**	343	Orange	20.82	20.93	21.92	22.18	23.43	23.65
**22h**	363	Orange	14.17	16.52	17.82	19.94	20.12	23.42

**Table 4:** Fastness properties of monoazo disperse dyes on polyester fabrics*.*

Dye	Dye o.m.f. %	Wash fastness *^a,b^*			Persipiration fastness						Light fastness
					Alkaline			Acedic			
		Alt	SC	SW	Alt	SC	SW	Alt	SC	SW	
**7**	**1%**	5	5	5	5	5	5	5	4	5	4
**22a**		5	5	5	5	5	5	5	5	5	3–4
**22b**		5	5	5	5	5	5	5	5	5	3
**22c**		5	5	5	5	5	5	5	5	5	3–4
**22d**		4–5	4–5	4–5	5	5	5	5	5	5	4
**13**		5	5	5	5	5	5	5	5	5	6
**22f**		5	5	5	5	5	5	5	5	5	3
**22g**		5	5	4–5	5	5	5	5	5	5	3
**22h**		5	5	5	5	5	5	5	5	5	6
**7**	**2%**	5	5	5	5	5	5	4–5	4	5	3–4
**22a**		5	5	5	5	5	5	5	5	5	3–4
**22b**		5	5	5	5	5	5	5	5	5	3
**22c**		5	5	5	5	5	5	4–5	4	5	3
**22d**		5	4	4–5	5	5	5	5	5	5	3
**13**		5	5	5	5	5	5	5	5	5	5–6
**22f**		5	5	4–5	5	5	5	5	5	5	2–3
**22g**		5	5	4–5	5	5	5	5	5	5	3
**22h**		5	5	5	5	5	5	5	5	5	6
**7**	**3%**	5	5	5	5	5	5	5	5	5	3–4
**22a**		5	5	5	5	5	5	5	5	5	3–4
**22b**		5	5	5	5	5	5	5	5	5	3–4
**22c**		5	5	5	5	5	5	5	5	5	3
**22d**		5	4–5	4–5	5	5	5	5	5	5	3
**13**		5	5	5	5	5	5	5	5	5	5
**22f**		5	5	4–5	5	4–5	5	5	5	5	2–3
**22g**		5	5	4–5	5	5	5	5	5	5	3
**22h**		5	5	5	5	5	5	5	5	5	6
**7**	**4%**	5	5	5	5	5	5	5	5	5	3
**22a**		5	5	5	5	5	5	5	5	5	3–4
**22b**		5	5	5	5	5	5	5	5	5	3–4
**22c**		5	5	5	5	5	5	5	5	5	3–4
**22d**		5	3–4	3–4	5	4–5	5	5	4–5	5	3
**13**		5	5	5	5	5	5	5	4–5	5	5–6
**22f**		5	5	4–5	5	5	5	5	5	5	2–3
**22g**		4–5	5	4–5	5	5	5	5	5	5	3–4
**22h**		5	5	5	5	5	5	5	5	5	6
**7**	**5%**	5	5	5	5	5	5	5	5	5	3–4
**22a**		5	5	5	5	5	5	5	5	5	3–4
**22b**		5	5	5	5	5	5	5	5	5	3–4
**22c**		5	5	5	5	5	5	5	5	5	4
**22d**		4–5	3–4	3–4	5	5	5	5	5	5	2
**13**		5	5	5	5	5	5	5	5	5	5–6
**22f**		5	5	4–5	5	5	5	5	5	5	2–3
**22g**		5	5	4–5	5	5	5	5	5	5	4
**22h**		5	5	5	5	5	5	5	5	5	6
**7**	**6%**	5	5	5	5	5	5	5	5	5	3
**22a**		5	5	5	5	5	5	5	5	5	4
**22b**		5	5	5	5	5	5	5	5	5	3–4
**22c**		5	5	5	5	5	5	5	5	5	4
**22d**		4–5	4	4	5	4–5	5	5	5	5	2
**13**		5	5	5	5	5	5	5	5	5	5–6
**22f**		5	5	4–5	4–5	4–5	4–5	5	4–5	5	2–3
**22g**		5	5	5	5	5	5	5	5	5	4
**22h**		5	5	5	5	5	5	5	5	5	6

*^a^* ISO CO2/CO41; *^b^* Alt = alteration; SC = staining on cotton; SW = staining on wool.

#### 2.2.1. Color Strength

The data given in Table 3 reveal that the color strengths (K/S) of the dyed polyester fabrics are directly proportional to the amounts of the dyes applied (% *o.m.f*.). The hues of the fabrics treated with the azo dyes were found to vary from yellowish-brown to orange in a manner that depends on the dye structures. Differences in the color strengths typically depend on substitution present in the arylazopyrazolopyrimidine disperse dyes [19,20,21]. The data in Table 3 clearly show that the magnitude of color strength obtained using dye **22d** is much larger than those for **22a**–**c** and **22f**–**h**. 

#### 2.2.2. Wash Fastness

The polyester samples, dyed with the disperse dyes **7**, **13**, **22a**–**d**, and **22f**–**h** using the high temperature dyeing method, were subjected to washing at 95 °C. The rates of leaching of the dyes from the fiber in the presence of soap (or synthetic detergent) solutions of various degrees of alkalinity are factors that determine fastness to washing. Table 4 showed that the washing fastness is excellent with respect to most of the tested compounds except compound **22d** (1%–6% shades) showed good results. Data showed that the magnitude of dye removal depends on the molecular size of the dye molecule, compound specific interactions between dye and substrate, wash liquor; molecular geometry; and concentration in substrate. 

#### 2.2.3. Light Fastness

The light fastness of each of the dyes was measured by employing the standard method for determination of color fastness of textiles. Several reports [22,23,24] suggest that fading of azo dyes is mainly a consequence of decomposition of the –N=N– moiety by either oxidation, reduction or photolysis. The rates of these processes should be sensitive to the chemical structure of the dye, the type of substrate and treatment conditions. Since the dyed substrate employed in this study is a polyester fabrics (*i.e.*, non-proteinic), the fading process likely occurs by oxidation [25]. The ease of oxidation of azo linkages should be a function of electron density. Therefore, electron donating substituents on this moiety should increase the fading rate while electron withdrawing groups should decrease the rate. This proposal is in agreement with the observed results (Table 4) which demonstrate that the presence of a methyl group in dyes **22b** and **22g** causes a decrease of light fastness to 3. On the other hand, the chlorine atom in dyes **22c** and **22h** is associated with an increase of light fastness to 4 and 6, respectively.

#### 2.2.4. Perspiration Fastness

The resistance to the action of human perspiration, referred to as the fastness to perspiration, is evaluated by treatment of a dyed material under alkaline and acidic conditions. The results of these tests (Table 4) using the dyed polyester indicate that the magnitude of dye removal under the influence of perspiration is dependent upon the more polar character of the dye molecule giving lower magnitudes of the dye removal (e.g., polyester fabrics dyed with **22h** have excellent perspiration fastness owing to the presence of the electronegative chlorine atom). In addition the results obtained showed that dyed fabrics have good fastness to perspiration may be due to: (a) the absence of solubilizing groups, which renders solubility, and wash ability of the dye-out of the fabrics, (b) the size of the dye molecule is considered relatively big, (c) the good intra-fiber diffusion of the dye molecules inside the fabrics.

## 3. Experimental

### 3.1. General

All melting points were recorded on a Gallenkamp apparatus and are uncorrected. IR spectra were recorded in KBr disks on a Perkin Elmer System 2000 FT-IR spectrophotometer. ^1^H- and ^13^C-NMR spectra were recorded on a Bruker DPX 400 MHz super-conducting NMR spectrometer. Mass spectra were measured on a VG Auto-spec-Q instrument (high resolution, high performance, tri-sector 5189 GC/MS/MS) and by LC MS using an Agilent 1100 series LC/MSD with API-ES/APCI ionization mode. Microanalyses were performed on a LECO CH NS-932 Elemental Analyzer. The microwave oven used is a single mode cavity Explorer Microwave (CEM Corporation, Matthews, NC, USA) and irradiate in heavy-walled Pyrex tube (capacity 10 mL and 80 mL for dyeing). The color strengths (K/S) of the dyed polyester fabrics and the color fastness to light were evaluated at the Dyeing, Printing and Textile Auxiliaries Department, Textile Research Division, National Research Centre (NRC), Giza, Egypt. X-ray crystallography was carried out on a Kappa CCD Enraf Nonius FR 590 diffractometer, National Research Centre, Giza, Egypt. 

### 3.2. General Procedure for the Synthesis of Azo Disperse Dyes

*4-(3,5-Diamino-1H-pyrazol-4-ylazo)-phenol *(**7**)*. *This compound was prepared according to the literature [6]. Mp 245–246 °C, ^1^H-NMR (DMSO-d_6_) δ 9.52 (s, 1H, OH), 7.52 (d, 2H, *J = *8.8 Hz), 6.77 (d, 2H, *J = *8.8 Hz), 6.46 (br, 1H, NH), 5.87 (br, 4H, 2NH_2_). ^13^C-NMR (DMSO-d_6_) δ 156.8, 146.4, 121.8, 115.5, 115.3, 115.2, 112.9. *λ*_max_ (DMF)/nm 369.

*2-[(4-Hydroxyphenyl)-hydrazono]-3-oxobutyronitrile *(**10**). *p*-Aminophenol (10.9 g, 0.1M) was dissolved in concentrated HCl (30 mL) and water (20 mL) cooled in ice and then NaNO_2_ (7 g) in water (50 mL) was added in portions. A mixture of 3-aminocrotononitrile (8.2 g, 0.1 mole), NaOAc (20 g), ethanol (15 mL), and water (50 mL) was prepared separately and cooled in ice. The diazonium salt solution was added slowly to the second solution, with ice cooling. The cooled reaction was stirred for 0.5 h and filtered to give brown crystals, which were crystallized from alcohol/water. Brown solid (7.0 g, 85%). Mp 214–215 °C. ^1^H-NMR (DMSO-d_6_) δ 12.18 (s, 1H, NH), 9.61 (s, 1H, OH), 7.39 (d, 1H, *J = *8.8 Hz), 6.81 (d, 1H, *J = *8.8 Hz), 2.37 (s, 3H, CH_3_). ^13^C-NMR (DMSO-d_6_) δ 192.6, 155.3, 134.1, 118.4, 115.9, 111.8, 111.3, 24.5. IR: 3199, 3073, 2922, 2213, 1631, 1599, 1465, 1440, 1367, 1329, 1286, 1268, 1226, 1208, 952, 835 cm^–1^. MS (EI) *m/z* (%) = 203 ([M]^+^, 100), 121 (53), 108 (93). HRMS: *m/z* (EI) for C_10_H_9_N_3_O_2_; calcd. 203.0689; found: 203.0689.

*4-(3-Amino-5-methyl-1H-pyrazol-4-ylazo)-phenol* (**13A**)* and 4-(5-Amino-3-methyl-1H-pyrazol-4-ylazo)-phenol *(**13B**). A mixture of **10** (2.03 g, 10 mmol), hydrazine hydrate (2.5 mL) in ethanol (20 mL) was stirred at reflux for 3–4 h. The solvent was removed under vacuum and the formed solid was collected and crystallized from ethanol/water. Brown solid (1.75 g, 86%). Mp 239–240 °C. ^1^H-NMR (DMSO-d_6_) δ 11.94 (br, 1H, NH, 13A), 11.51 (br, 1H, NH, 13B), 9.92 (s, 2H, 2OH, 13A,13B), 7.56 (d, 4H, *J *= 8.4 Hz, 13A,13B), 6.81 (d, 4H, *J *= 8.4 Hz, 13A,13B), 6.75 (br, 2H, NH_2_), 5.84 (br, 2H, NH_2_), 2.36 (s, 6H, 2CH_3_). ^13^C-NMR (DMSO-d_6_) δ 157.9, 148.2, 146.0, 140.6, 122.3, 115.5, 115.2, 18.5. *λ*_max_ (DMF)/nm 376. IR: 3399, 3267, 3213, 1629, 1590, 1530, 1461, 1386, 1256, 1095, 838 cm^–1^. MS (EI) *m/z* (%) =217 ([M]+, 100), 124 (60), 93 (12). HRMS: *m/z* (EI) for C_10_H_11_N_5_O; calcd. 217.0958; found: 217.0958. 

### 3.3. Synthesis of Compounds **14a–b**, **20a,b** and **22a–j**

General procedure: a mixture of **7** (0.218 g, 1 mmol) or **13** (0.217 g, 1 mmol), acetylacetone, 2-piperidinylacrylonitrile or enaminones **21a**–**e** (1 mmol) in acetic acid (20 mL) and sod. acetate (0.12 g, 1.5 mmol) was refluxed for 1 h. The reaction mixture was poured onto ice water (50 mL) filtered and crystallized from the DMF solvent.

*4-(2-Amino-5,7-dimethyl-pyrazolo[1,5-a]pyrimidin-3-ylazo)-phenol* (**14a**). Yellow solid (0.22 g, 77%). Mp 287–288 °C. ^1^H-NMR (DMSO-d_6_) δ 9.81 (s, 1H, OH), 7.65 (dd, 2H, *J* = 7.2 and 1.8 Hz), 7.05 (br, 2H, NH_2_), 6.89 (s, 1H), 6.85 (dd, 2H, *J* = 7.2 and 1.8 Hz), 2.57 (s, 3H), 2.52 (s, 3H). ^13^C-NMR (DMSO-d_6_) δ 159.7, 158.2, 151.6, 146.1, 145.9, 145.0, 122.7, 115.6, 113.7, 109.1, 24.1, 16.4. IR: 3414, 3281, 1624, 1564, 1444, 1360, 1200, 1138, 837 cm^−1^. MS (EI) *m/z* (%) = 282 ([M]+, 100), 189 (40), 161 (20). HRMS: *m/z* (EI) for C_14_H_14_N_6_O; calcd. 282.1223; found: 282.1223.

*4-(2,5,7-Trimethylpyrazolo[1,5-a]pyrimidin-3-ylazo)-phenol* (**14b**). Greenish yellow solid (0.20 g, 72%). Mp > 350 °C. ^1^H-NMR (DMSO-d_6_) δ 9.98 (s, 1H, OH), 7.68 (dd, 2H, *J* = 7.2 and 1.8 Hz), 7.04 (s, 1H), 6.89 (dd, 2H, *J* = 7.2 and 1.8 Hz), 2.70 (s, 3H), 2.66 (s, 3H), 2.59 (s, 3H). ^13^C-NMR (DMSO-d_6_) δ 161.4, 159.1, 146.8, 146.4, 145.7, 144.0, 124.5, 123.3., 115.6, 110.1, 24.4, 16.2, 15.3. IR: 3427, 3061, 1617, 1596, 1562, 1495, 1448, 1405, 1370, 1263, 1116, 849 cm^−1^. MS (EI) *m/z* (%) = 281 ([M]^+^, 100), 188 (90), 160 (50). HRMS: *m/z* (EI) for C_15_H_15_N_5_O; calcd. 281.1271; found: 281.1271.

*4-(2,7-Diaminopyrazolo[1,5-a]pyrimidin-3-ylazo)-phenol* (**20a**). Reddish brown solid (0.19 g, 70%). Mp 248–249 °C. ^1^H-NMR (DMSO-d_6_) δ 9.76 (s, 1H, OH), 8.02 (d, 1H, *J = *5.6 Hz), 7.68 (br, 2H, NH_2_), 7.61 (d, 2H, *J* = 8.8 Hz), 6.89 (br, 2H, NH_2_), 6.84 (d, 2H, *J *= 8.8 Hz), 6.20 (d, 1H, *J *= 5.6 Hz) ^13^C-NMR (DMSO-d_6_) δ 157.8, 151.2, 149.5, 146.9, 146.7, 146.2, 122.3, 115.5, 113.8, 91.5. IR: 3456, 3330, 2972, 1632, 1485, 1452, 1373, 1159, 954, 835 cm^−1^. MS (EI) *m/z* (%) = 269 ([M]^+^, 100), 176 (33), 148 (30). HRMS: *m/z* (EI) for C_12_H_11_N_7_O; calcd. 269.1019; found: 269.1019.

*4-(7-Amino-2-methylpyrazolo[1,5-a]pyrimidin-3-ylazo)-phenol* (**20b**). Brown solid (0.20 g, 75%). Mp 291–292 °C. ^1^H-NMR (DMSO-d_6_) δ 9.89 (s, 1H, OH), 8.18 (d, 1H, *J *= 5.4 Hz), 8.00 (br, 2H), 7.64 (dd, 2H, *J* = 7.6 and 1.8 Hz), 6.87 (dd, 2H, *J* = 7.6 and 1.8 Hz), 6.25 (d, 1H, *J *= 5.4 Hz) 2.66 (s, 3H). ^13^C-NMR (DMSO-d_6_) δ 158.6, 151.5, 148.2, 147.8, 146.7, 144.3, 124.1, 122.9, 115.6, 90.8, 14.7. IR: 3476, 3347, 3182, 1639, 1596, 1481, 1450, 1369, 1322, 1275, 1251, 1145, 828 cm^−1^. MS (EI) *m/z* (%) = 268 ([M]^+^, 100), 175 (55), 147 (40). HRMS: *m/z* (EI) for C_13_H_12_N_6_O; calcd. 268.1067; found: 268.1067*.*

*4-(2-Amino-7-phenylpyrazolo[1,5-a]pyrimidin-3-ylazo)-phenol* (**22a**). Orange solid (0.25 g, 76%). Mp 301–302 °C. ^1^H-NMR (DMSO-d_6_) δ 9.88 (s, 1H, OH), 8.58 (d, 1H, *J *= 4.8 Hz), 8.08 (m, 2H,), 7.71 (d, 2H, *J* = 8.8 Hz) 7.61 (m, 3H), 7.21 (d, 1H, *J *= 4.8 Hz), 7.14 (br, 2H, NH_2_), 6.87 (d, 2H, *J* =8.8 Hz). ^13^C-NMR (DMSO-d_6_) δ 158.5, 151.8, 150.3, 147.3, 146.1, 144.9, 130.9, 130.5, 129.5, 128.5, 122.9, 115.6, 113.9, 108.6. *λ*_max_ (DMF)/nm 385. IR: 3432, 3318, 3167, 1627, 1550, 1450, 1271, 1242, 832 cm^−1^. MS (EI) *m/z* (%) = 330 ([M]^+^, 100), 237 (25), 115 (30). Anal. calcd. for C_18_H_14_N_6_O (330.3): C 65.44; H 4.27; N 25.44. Found: C 65.34; H 4.20; N 25.37.

*4-(2-Amino-7-p-methylphenylpyrazolo[1,5-a]pyrimidin-3-ylazo)-phenol* (**22b**). Reddish brown solid (0.29 g, 84%). Mp 309–310 °C. ^1^H-NMR (DMSO-d_6_) δ 9.88 (s, 1H, OH), 8.54 (d, 1H, *J *= 4.4 Hz), 8.02 (d, 2H, *J = *8.4 Hz), 7.72 (d, 2H, *J* = 8.8 Hz), 7.41 (d, 2H, *J* = 8.4 Hz), 7.19 (d, 1H, *J = *4.4 Hz), 7.14 (br, 2H, NH_2_), 6.88 (d, 2H, *J *= 8.8 Hz), 2.43 (s, 3H). ^13^C-NMR (DMSO-d_6_) δ 158.4, 151.8, 150.1, 147.3, 146.1, 144.9, 141.1, 129.4, 129.0, 127.5, 122.8, 115.6, 113.8, 108.2, 21.1. *λ*_max_ (DMF)/nm 385. IR: 3421, 3309, 2917, 1602, 1548, 1447, 1328, 1232, 1095, 833 cm^−1^. MS (EI) *m/z* (%) = 344 ([M]^+^, 80), 196 (30), 129 (100). HRMS: *m/z* (EI) for C_19_H_16_N_6_O; calcd. 344.1380; found: 344.1380.

*4-(2-Amino-7-p-chlorophenylpyrazolo[1,5-a]pyrimidin-3-ylazo)-phenol* (**22c**). Orange solid (0.28 g, 78%). Mp 306–307 °C. ^1^H-NMR (DMSO-d_6_) δ 9.89 (s, 1H, OH), 8.58 (d, 1H, H^5^, *J *= 4.4 Hz), 8.14 (d, 2H, H^9^, *J *= 8.4 Hz), 7.72 (d, 2H, H^13^, *J* = 8.8 Hz) 7.69 (d, 2H, H^10^, *J* = 8.4 Hz), 7.24 (d, 1H, H^6^, *J *= 4.4 Hz), 7.17 (br, 2H, NH_2_), 6.88 (d, 2H, H^14^, *J* = 8.8 Hz). ^13^C-NMR (DMSO-d_6_) δ 158.5, 151.8, 150.2, 147.2, 146.1, 143.7, 135.7, 131.3, 129.2, 128.5, 122.8, 115.6, 113.9, 108.4. * λ*_max_ (DMF)/nm 383. IR: 3418, 3308, 3180, 1616, 1601, 1549, 1487, 1450, 1331, 1271, 1237, 1088, 842 cm^−1^. MS (EI) *m/z* (%) = 364 ([M]^+^, 100), 271 (32), 149 (42). HRMS: *m/z* (EI) for C_18_H_13_ClN_6_O; calcd. 364.0833; found: 364.0833.

*4-(2-Amino-7-furanpyrazolo[1,5-a]pyrimidin-3-ylazo)-phenol* (**22d**). Red solid (0.26 g, 80%). Mp 292–293 °C. ^1^H-NMR (DMSO-d_6_) δ 9.87 (s, 1H, OH), 8.55 (d, 1H, *J* = 5.2), 8.19 (m, 2H), 7.71 (dd, 2H, *J* = 7.2 and 1.6), 7.43 (d, 1H*J* = 5.2), 7.24 (br, 2H, NH_2_), 6.94 (dd, 1H, *J* = 3.6,1.6), 6.87 (dd, 2H, *J* 7.2 and 1.6). ^13^C-NMR (DMSO-d_6_) δ 158.5, 151.9, 149.3, 147.4, 146.9, 146.0, 143.0, 133.8, 122.9, 120.0, 115.6, 113.5, 113.3, 103.1. *λ*_max_ (DMF)/nm 378. IR: 3449, 3411, 2923, 1595, 1449, 1316, 1009, 824 cm^−1^. MS (EI) *m/z* (%) = 320 ([M]^+^, 100), 227 (30), 116 (15). HRMS: *m/z* (EI) for C_16_H_12_N_6_O_2_ calcd. 320.1016; found: 320.1016.

*4-(2-Amino-7-thiophenepyrazolo[1,5-a]pyrimidin-3-ylazo)-phenol* (**22e**). Brown solid (0.27 g, 80%). Mp 276–277 °C. ^1^H-NMR (DMSO-d_6_) δ 9.93 (s, 1H, OH), 8.54 (d, 1H, *J* = 3.6), 8.52 (d, 1H, *J* = 4.8), 8.16 (dd, 1H, *J* = 4.8 and 1.2), 7.73 (m, 3H), 7.40 (dd, 1H, *J* = 4.8 and 1.2), 7.30 (br, 2H, NH_2_), 6.88 (d, 2H, *J* = 8.8). ^13^C-NMR (DMSO-d_6_) δ 158.7, 151.5, 149.2, 147.1, 145.9, 138.2, 134.9, 132.4, 129.9, 128.0, 122.8, 115.7, 113.5, 104.5. *λ*_max_ (DMF)/nm 370, IR: 3437, 3107, 3023, 1602, 1549, 1500, 1450, 1414, 1330, 1242, 1183, 839 cm^−1^. MS (EI) *m/z* (%) = 336 ([M]^+^, 100), 243 (50), 188 (20). Anal. calcd. for C_16_H_12_N_6_OS (336.4): C 57.13; H 3.60; N 24.98; S 9.53. Found: C 57.04; H 3.48; N 24.87; S 9.48.

*4-(2-Methyl-7-phenylpyrazolo[1,5-a]pyrimidin-3-ylazo)-phenol* (**22f**). Yellow solid (0.28 g, 85%). Mp 297–298 °C. ^1^H-NMR (DMSO-d_6_) δ 10.05 (s, 1H, OH), 8.79 (d, 1H, *J *= 4.2 Hz), 8.11 (m, 2H), 7.73 (dd, 2H, = *J* 8.4 and 1.8 Hz), 7.65 (m, 3H), 7.38(d, 1H, *J *= 4.2 Hz), 6.92 (dd, 2H, *J *= 8.4 and 1.8 Hz), 2.68 (s, 3H).^13^C-NMR (DMSO-d_6_) δ 159.5, 152.1, 147.8, 146.4, 144.7, 144.6, 136.1, 131.5, 128.8, 128.6, 125.1, 123.5, 115.7, 109.4, 14.9. *λ*_max_ (DMF)/nm 372. IR: 3163, 3066, 1587, 1542, 1482, 1331, 1228, 1263, 1083, 819 cm^−1^. MS (EI) *m/z* (%) = 329 ([M]^+^, 100), 236 (70), 182 (30). Anal. calcd. for C_19_H_15_N_5_O (329.4): C 69.29; H 4.59, N 21.26. Found: C 69.27; H 4.67; N 21.25. 

*4-(2-Methyl-7-p-methylphenylpyrazolo[1,5-a]pyrimidin-3-ylazo)-phenol* (**22g**). Reddish brown solid (0.28 g, 83%). Mp 267–268 °C. ^1^H-NMR (DMSO-d_6_) δ 10.01 (s, 1H, OH), 8.73 (d, 1H, *J *= 4.4 Hz), 8.03 (d, 2H, *J = *8.0 Hz), 7.70 (d, 2H, *J* = 8.8 Hz), 7.42 (d, 2H, *J *= 8.0 Hz), 7.33 (d, 1H, *J *= 4.4 Hz), 6.89 (d, 2H, *J *= 8.8 Hz), 2.66 (s, 3H), 2.42 (s, 3H). ^13^C-NMR (DMSO-d_6_) δ 159.9, 152.5, 148.2, 147.0, 146.4, 145.3, 141.9, 130.1, 129.6, 127.6, 125.5, 123.9, 116.2, 109.5, 21.6, 15.5. *λ*_max_ (DMF)/nm 373. IR: 3418, 3144, 2923, 1602, 1542, 1502, 1274, 1143, 841cm^−1^. MS (EI) *m/z* (%) = 343 ([M]^+^, 100), 250 (53), 196 (33). HRMS: *m/z* (EI) for C_20_H_17_N_5_O; calcd. 343.1428; found: 343.1427.

*4-(2-Methyl-7-p-chlorophenylpyrazolo[1,5-a]pyrimidin-3-ylazo)-phenol* (**22h**). Orange solid (0.29 g, 80%). Mp 259–260 °C. ^1^H-NMR (DMSO-d_6_) δ 10.06 (s, 1H, OH), 8.79 (d, 1H, *J *= 4.2 Hz), 8.16 (d, 2H, *J* = 8.8 Hz), 7.72 (m, 4H), 7.4 (d, 1H, *J *= 4.2 Hz), 6.92 (d, 2H, *J *= 8.8 Hz), 2.68 (s, 3H).^13^C-NMR (DMSO-d_6_) δ 159.4, 152.1, 147.8, 146.5, 145.9, 144.7, 131.2, 130.1, 129.6, 128.6, 125.0, 123.4, 115.7, 109.4, 14.9. *λ*_max_ (DMF)/nm 373. IR: 3430, 3162, 3053, 1598, 1548, 1491, 1452, 1353, 1269, 1241, 1145, 836 cm^−1^. MS (EI) *m/z* (%) = 363 ([M]^+^, 100), 270 (75), 216 (30). Anal. calcd. for C_19_H_14_ClN_5_O (363.8): C 62.73; H 3.88, N 19.25. Found: C 62.90; H 3.87; N 19.30. 

*4-(2-Methyl-7-furanepyrazolo[1,5-a]pyrimidin-3-ylazo)-phenol* (**22i**). Red crystals were obtained from DMF, (0.25 g, 78%). Mp 284–285 °C. ^1^H-NMR (DMSO-d_6_) δ 10.06 (s, 1H, OH), 8.76 (d, 1H, *J* = 4.8 Hz), 8.21 (m, 2H), 7.72 (dd, 2H, *J* = 7.0 and 1.8 Hz), 7.56 (d, 1H, *J* = 4.8 Hz), 6.93 (m, 1H), 6.91 (dd, 2H, *J* = 7.0 and 1.8 Hz), 2.76 (s, 3H). ^13^C-NMR (DMSO-d_6_) δ 159.5, 151.3, 148.0, 147.7, 146.5, 144.5, 142.7, 134.7, 124.9, 123.5, 120.4, 115.7, 113.5, 104.0, 15.1. *λ*_max_ (DMF)/nm 373. IR: 3157, 3035, 2978, 1596, 1569, 1522, 1450, 1350, 1265, 1236, 1145, 1015, 842 cm^−1^. MS (EI) *m/z* (%) = 319 ([M]^+^, 100), 226 (45), 198 (30). Anal. calcd. for C_17_H_13_N_5_O_2_ (319.3): C 63.94; H 4.10, N 21.93. Found: C 63.93; H 4.08; N 21.78.

*4-(2-Methyl-7-thiophenepyrazolo[1,5-a]pyrimidin-3-ylazo)-phenol* (**22j**). Reddish brown solid (0.24 g, 72%). Mp 278–279 °C. ^1^H-NMR (DMSO-d_6_) δ 10.04 (s, 1H, OH), 8.73 (d, 1H, *J* = 4.8 Hz), 8.57 (dd, 1H, *J* = 4.2 and 1.2 Hz), 8.15 (dd, 1H, *J* = 4.8 and 1.2 Hz), 7.86 (d, 1H, *J* = 4.8 Hz), 7.72 (dd, 2H, *J* = 7.2 and 1.8 Hz), 7.40 (dd, 1H, , *J* = 4.6 and 3.6 Hz), 6.92(dd, 2H, *J* = 7.2 and 1.8 Hz), 2.76 (s, 3H). ^13^C-NMR (DMSO-d_6_) δ 159.4, 151.2, 147.7, 146.5, 144.5, 139.2, 135.3, 132.8, 129.5, 127.8, 124.9, 123.5, 115.7, 105.4, 15.1. *λ*_max_ (DMF)/nm 376, IR: 3407, 3097, 2972, 1592, 1542, 1500, 1448, 1330, 1237, 1145, 1008, 843 cm^−1^. MS (EI) *m/z* (%) = 335 ([M]^+^, 100), 242 (70), 188 (30). Anal. calcd. for C_17_H_13_N_5_OS (335.4): C 60.88; H 3.91, N 20.88; S 9.56. Found: C 61.01; H 3.92; N 21.01; S 9.44.

### 3.4. General Procedure for the Preparation of **30a–b**

A mixture of **7 **(0.218 g, 1 mmol) or **13** (0.217 g, 1 mmol) in ethanol (20 mL), 5 drops of piperidine, and benzylidenemalononitrile (0.154 g, 1 mmol) was refluxed for 6 h, then the reaction mixture was poured into ice water, and neutralized by HCl, filtered off and recrystallized from ethanol. 

*2,5-Diamino-3-(4-hydroxyphenylazo)-7-phenylpyrazolo[1,5-a]pyrimidine-6-carbonitrile *(**30a**)*. *Reddish brown solid (0.22 g, 60%). Mp 195–196 °C. ^1^H-NMR (DMSO-d_6_) δ 9.87 (br, s, 1H, OH), 8.50 (br, 2H, NH_2_), 7.86 (dd, 2H, *J* = 8.4 and 1.4 Hz), 7.68 (d, 2H, *J* = 8.8 Hz), 7.57–7.55 (m, 3H), 6.98 (br, 2H, NH_2_), 6.86 (d, 2H, *J* = 8.8). ^13^C-NMR (DMSO-d_6_) δ 160.5, 158,8, 152.3, 148.9, 145.9, 145.5, 137.4, 130.2, 128.7, 128.3, 123.1, 116.4, 115.9, 115.7, 74.6. IR: 3429, 2919, 2850, 2213, 1624, 1471, 1367, 1130, 844 cm^−1^. MS (EI) *m/z* (%) = 370 ([M]^+^, 90), 321 (100), 265 (23). HRMS: *m/z* (EI) for C_19_H_14_N_8_O; calcd. 370.1285; found: 370.1285.

*5-Amino-3-(4-hydroxyphenylazo)-2-methyl-7-phenylpyrazolo[1,5-a]pyrimidine-6-carbonitrile *(**30b**). Yellow solid (0.23g, 63%). Mp 302–303 °C. ^1^H-NMR (DMSO-d_6_) δ 9.97 (br, s, 1H, OH), 9.00 (br, 2H, NH_2_), 7.87 (dd, 2H, *J* = 8.4 and 1.4 Hz), 7.67(d, 2H, *J* = 8.4 Hz), 7.57–7.54 (m, 3H), 6.89 (d, 2H, *J* = 8.4), 2.68 (s, 3H, CH_3_). ^13^C-NMR (DMSO-d_6_) δ 160.9, 159.5, 150.4, 148.7, 146.3, 143.9, 137.3, 130.2, 128.7, 128.3, 126.1, 123.5, 116.1, 115.7, 74.3, 15.5. IR: 3431, 3304, 3235, 3166, 2213, 1643, 1593, 1449, 1287, 1148, 842 cm^−1^. MS (EI) *m/z* (%) = 369 ([M]^+^, 100), 276 (43), 222 (18). HRMS: *m/z* (EI) for C_20_H_15_N_7_O; calcd. 369.1332; found: 369.1332.

### 3.5. High Temperature Dyeing Method (HT)

#### 3.5.1. Materials

Scoured and bleached 100% polyester (150, 130 g/m^2^, 70/2 denier) was obtained from El-Shourbagy Co., Cairo, Egypt. The fabric was treated before dyeing with a solution containing nonionic detergent (Hostapal CV, Clariant-Egypt, 5 g/L) and sodium carbonate (2 g/L) in a ratio of 50:1 at 60 °C for 30 min, thoroughly washed with water, and air dried at room temperature.

#### 3.5.2. Dyeing

A dispersion of the dye was produced by dissolving the appropriate amount of dye (1%–6% shades) in 1 cm^3^ acetone and then added dropwise with stirring to the dyebath (liquor ration 20:1) containing sodium lignin sulphonate as dispersing agent. The pH of the dyebath was adjusted to 5.5 using aqueous acetic acid and the wetted-out polyester fabrics were added. Dyeing was performed by raising the dyebath temperature to 130 °C under pressure in a microwave oven at a rate of 20 °C/min, holding at this temperature for 60 min and rapidly cooling to 50 °C. After dyeing, the fabrics were thoroughly washed and subjected to surface reduction clearing [(5 g NaOH + 6 g sodium hydrosulphite)/L]. The samples were heated in this solution for 10 min at 60 °C and then thoroughly washed and air-dried.

### 3.6. Color Measurements and Analyses

#### 3.6.1. Color Measurements of the Dyed Fabrics

The color yields of the dyed samples were determined by using the light reflectance technique performed on a Perkin-Elmer (Lambda 3B) UV/VIS Spectrophotometer. The color strengths, expressed as K/S values, were determined by applying the Kubelka-Mink equation:

K/S = [(1 − R)^2^/2R] − [(1 − R_o_)^2^/2R_o_]

where *R *= decimal fraction of the reflectance of the dyed fabric, *R_o_* = decimal fraction of the reflectance of the undyed fabric, *K* = absorption coefficient, and *S* = scattering coefficient.

#### 3.6.2. Color Fastness Tests

##### 3.6.2.1. Fastness to Washing

After washing using 2 g/L of the nonionic detergent Hostapal CV at 80 °C for 15 min, the dyed fabrics were tested by using ISO standard methods [26]. A specimen of dyed polyester fabric was stitched between two pieces of undyed cotton and wool fabrics, all of equal length, and then washed at 95 °C for 30 min. The staining on the undyed adjacent fabrics was assessed according to the following gray scale: 1—poor, 2—fair, 3—moderate, 4—good, 5—excellent.

##### 3.6.2.2. Fastness to Perspiration

The samples were prepared by stitching a piece of dyed polyester fabric between two pieces of cotton and wool fabrics, all of equal length, and then immersed in the acid or alkaline solution for 30 min. The staining on the undyed adjacent fabrics was assessed according to the following gray scale: 1—poor, 2—fair, 3—moderate, 4—good, 5—excellent. The acid solution (pH = 3.5) contains sodium chloride (10 g/L), sodium dihydrogen orthophosphate (1 g/L) and histidine monohydrochloride (0.25 g/L). The alkaline solution (pH = 8) contains sodium chloride (10 g/L), disodium orthophosphate (1 g/L) and histidine monohydrochloride (0.25 g/L).

##### 3.6.2.3. Fastness to Light

Light fastness was determined by exposing the dyed polyester fabrics on a Xenotest 150 (Original Hanau, city, country). Chamber temperature: 25–30 °C, black panel temperature: 60 °C, relative humidity: 50–60%, dark glass UV filter system) for 40 h. The changes in color were assessed according to the following blue scale: 1—poor, 3—moderate, 4—good, 6—very good, 8—excellent.

## 4. Conclusions

This study describes the synthesis of some new monoazo disperse pyrazolopyrimidine dyes, which involves initial coupling of malononitrile or 3-aminocrotononitrile with 4-hydroxybenzenediazonium chlorides, subsequent treatment of the hydrazone products with hydrazine hydrate gave the corresponding 4-hydroxyphenylazoaminopyrazoles that were then treated with either 2,4-pentandione or arylenaminoketones to give the target pyrazolopyrimidine monoazo disperse dyes. The dyes produced in this manner were then applied to polyester fabrics using the HT dyeing method assisted by microwave irradiation. The dyed fabrics, which display yellowish brown to orange hues, were found to have moderate fastness to light and excellent fastness levels to washing and perspiration.
